# Investigation of 3.3 kV 4H-SiC DC-FSJ MOSFET Structures

**DOI:** 10.3390/mi12070756

**Published:** 2021-06-27

**Authors:** Chia-Yuan Chen, Yun-Kai Lai, Kung-Yen Lee, Chih-Fang Huang, Shin-Yi Huang

**Affiliations:** 1 Department of Engineering Science and Ocean Engineering, National Taiwan University, Taipei 10617, Taiwan; r08525070@ntu.edu.tw (C.-Y.C.); r07525008@ntu.edu.tw (Y.-K.L.); 2 Advanced Research Center for Green Materials Science and Technology, National Taiwan University, Taipei 10617, Taiwan; 3 Institute of Electronics Engineering, National Tsing Hua University, Hsinchu 300044, Taiwan; cfhuang@ee.nthu.edu.tw; 4 Industrial Technology Research Institute, Hsinchu 310401, Taiwan; Sandy@itri.org.tw

**Keywords:** silicon carbide, superjunction, breakdown voltage, specific on-resistance, MOSFET, 4H-SiC

## Abstract

This research proposes a novel 4H-SiC power device structure—different concentration floating superjunction MOSFET (DC-FSJ MOSFET). Through simulation via Synopsys Technology Computer Aided Design (TCAD) software, compared with the structural and static characteristics of the traditional vertical MOSFET, DC-FSJ MOSFET has a higher breakdown voltage (BV) and lower forward specific on-resistance (R_on,sp_). The DC-FSJ MOSFET is formed by multiple epitaxial technology to create a floating P-type structure in the epitaxial layer. Then, a current spreading layer (CSL) is added to reduce the R_on,sp_. The floating P-type structure depth, epitaxial layer concentration and thickness are optimized in this research. This structure can not only achieve a breakdown voltage over 3300 V, but also reduce R_on,sp_. Under the same conditions, the Baliga Figure of Merit (BFOM) of DC-FSJ MOSFET increases by 27% compared with the traditional vertical MOSFET. R_on,sp_ is 25% less than that of the traditional vertical MOSFET.

## 1. Introduction

Currently, most power devices are based on mature Si technology and applied to the high voltage areas. As the material properties of silicon materials are restricted by higher breakdown voltage, operating temperature and switching frequency, a Si IGBT seems to be the better option for higher power systems. However, the switching speed of a Si IGBT is not as fast as that of a Si power MOSFET, so it is difficult for the silicon power devices to achieve higher power conversion efficiency. Instead, wide band gap materials (such as silicon carbide, gallium nitride and gallium oxide) are very suitable for the applications of high power. In terms of physical characteristics, silicon carbide materials have the advantages of a wider band gap, a higher breakdown electric field and higher thermal conductivity [1,2,3,4,5] compared with silicon materials. Therefore, many SiC power devices are developed and applied in various areas.

In general, power MOSFET structures can be divided into lateral double-implanted metal oxide semiconductor field-effect transistors (LDMOSFET) and vertical double-implanted metal oxide semiconductor field-effect transistors (VDMOSFET). In the design of high power devices, an LDMOSFET needs a wider drift region in order to withstand high voltage so that the chip size is larger; a VDMOSFET needs a thicker drift region to maintain higher voltage so that the chip size can be smaller and the problem of the excessive surface electric field can also be improved. Therefore, when designing high voltage devices, the vertical device structure is a better choice.

As the demand of breakdown voltage is increased, the resistances of the JFET region and drift region inside a power MOSFET increase rapidly [6,7,8,9,10,11,12,13], causing power dissipation. Therefore, it is necessary to develop a power MOSFET with low resistance. To date, 650, 1200 and 1700 V SiC MOSFETs with relatively low specific on-resistance are already commercialized, while 3300 V SiC MOSFET products will be commercialized soon.

This research proposes a floating structure that can reach a breakdown voltage greater than 3300 V and reduce the R_on,sp_. Compared with the traditional whole-column superjunction structure which needs more epitaxial regrowth, the floating-P structure can not only achieve the same result, but also reduce the time and cost of the epitaxial regrowth. In addition, the challenges in fabricating the superjunction MOSFET are the ability to precisely control the concentration and the uniform thickness of each epilayer. It will increase the difficulty of fabrication if more concentrations of the drift region are used. Therefore, two concentrations of the drift region are used.

## 2. Structures of MOSFETs

**Figure 1:**
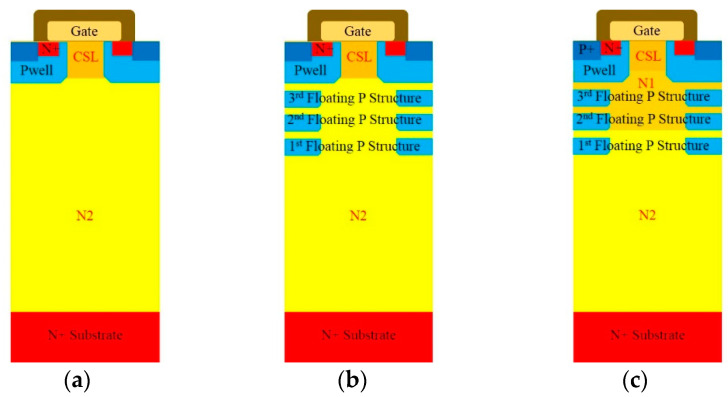
Three device structure schematic diagrams (**a**) traditional vertical MOSFET, (**b**) FSJ MOSFET, (**c**) DC-FSJ MOSFET.

Technology Computer Aided Design (TCAD) software (Sentaurus, Synopsys, Mountain View, CA, USA), is used to simulate structures and performances of 3.3 kV 4H-SiC MOSFETs. The three devices are a traditional vertical MOSFET, a MOSFET with three floating P-type structure and a MOSFET with three floating P-type structures and two different concentrations of the drift region, known as the floating superjunction MOSFET (FSJ MOSFET) and the different concentration floating superjunction MOSFET (DC-FSJ MOSFET), respectively. DC-FSJ MOSFET is the main structure designed in this paper. Figure 1a shows a schematic diagram of a traditional vertical MOSFET with an epitaxial layer thickness of 30 μm. The concentration and thickness are commonly used in academia and the industry for 3.3 kV 4H-SiC MOSFET. When the V_DS_ (Drain-source voltage) is applied and the V_GS_ (Gate-source voltage) is larger than the threshold voltage, the MOSFET will be turned on and the current will vertically flow through the MOSFET from the drain to the source. When the V_GS_ is smaller than the threshold voltage and the V_DS_ is still applied, the MOSFET will be turned off. There will be no more current, the MOSFET will be then depleted and the electric field is induced. If the electric field is larger than the critical value, the MOSFET will break down. Therefore, the structure and parameters need to be well designed [14,15,16,17,18,19,20,21,22,23]. Figure 1b shows a schematic diagram of a MOSFET with three floating P-type structures. Considering the limitation and cost of producing 4H-SiC SJ MOSFET via multiple epitaxy growth, the more floating P-type structures, the longer the process is. The depth and spacing of the three floating P-type structures are the same. The thickness of each epitaxial layer is 2 μm and the thickness of the total epitaxial layer is 30 μm. The concentrations of the three floating P-type structures are the same. The uppermost layer (the JEFT region) is the CSL, which can improve the current spreading ability when the device is turned on and reduce the parasitic JFET effect [14,15,16]. Figure 1c is DC-FSJ MOSFET. The thickness of the epitaxial layer is 26 μm, which enables the device to maintain a breakdown voltage greater than 3800 V and also reduce the R_on,sp_. The epitaxial layer is divided into three layers; the top layer is CSL and the other epitaxial layers under the CSL are N1 and N2 epitaxial layers. The concentration of the N1 epitaxial layer is higher than that of the N2 epitaxial layer. The purpose of the N1 epitaxial layer is to achieve the charge compensation with the floating P-type structures [17,18,19]. In addition to maintaining the breakdown voltage of the MOSFET, it can also greatly reduce R_on,sp_ of the device. In order to withstand most of the reverse voltage, the N2 epitaxial layer concentration is relatively lighter. The main differences between the three MOSFET structures are the floating P-type structures and the N1 epitaxial layer.

## 3. Influence of Structure and Doping

### 3.1. Floating P-Type Structure Depth

**Figure 2:**
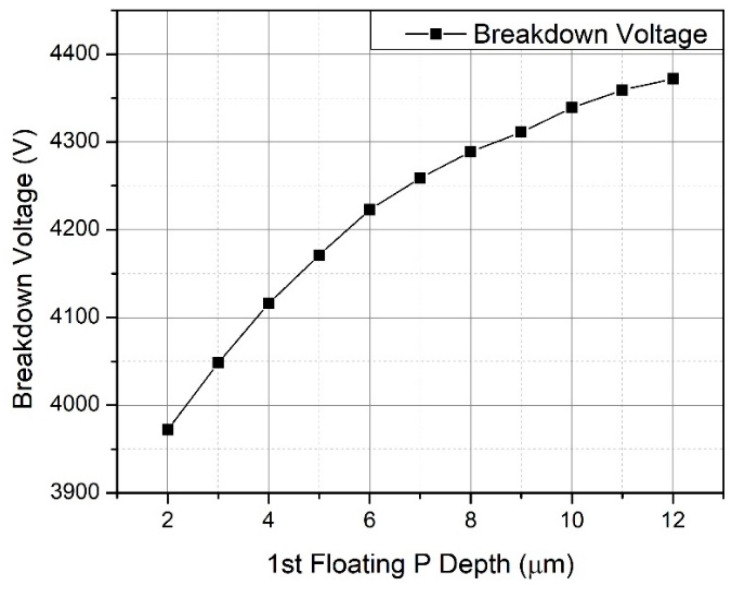
Relationship of different floating P-type structure depths and breakdown voltage.

**Figure 3:**
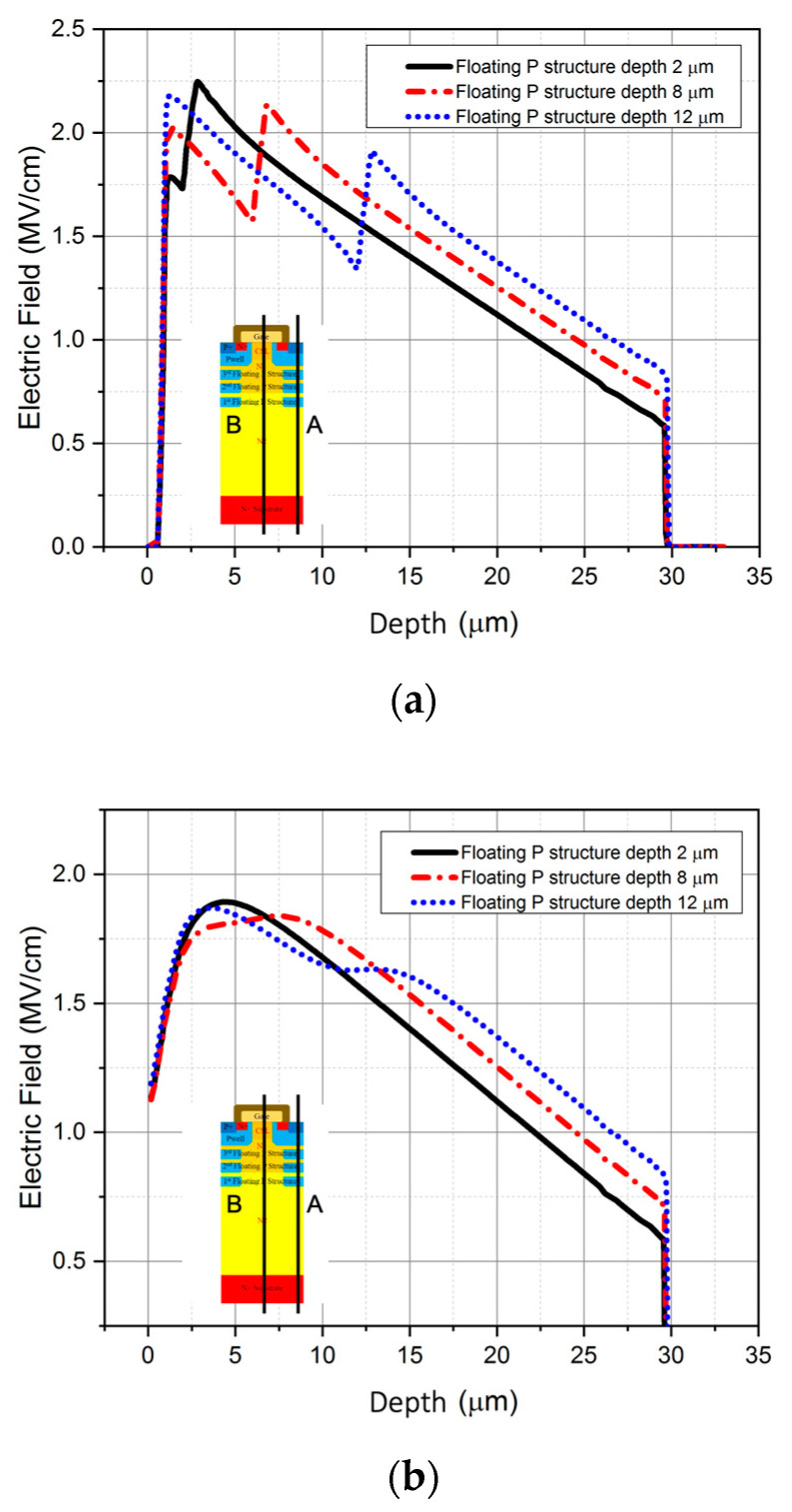
The electric field distribution of different floating P-type structure depths (**a**) in the P-well region and the floating P-type structure area, (**b**) in the drift region of the MOSFET.

For designing the depth of the P-type structure, the deepest floating P-type structure is investigated in the beginning. The simulation results show that the breakdown voltage of the MOSFET increases as the floating P-type structure becomes deeper in the epitaxial layer. By changing the depth of the floating P-type structure, the electric field extension is enhanced so that the breakdown voltage increases [20,21,22], as shown in Figure 2. However, when the depth of the floating P-type structure exceeds 8 μm, as seen from the vertical electric field distribution, the electric field along the PN region shown in Figure 3a and MOS region shown in Figure 3b becomes uneven, leading to the slightly increasing breakdown voltage; this is because the distance between the structure and the P-well is too long.

Considering the multiple epitaxial growth, the depth of the floating P-type structure, process time and alignment deviation, a depth of 6 μm and three P-type structures are chosen.

### 3.2. Concentration of N1 Epitaxial Layer

**Figure 4:**
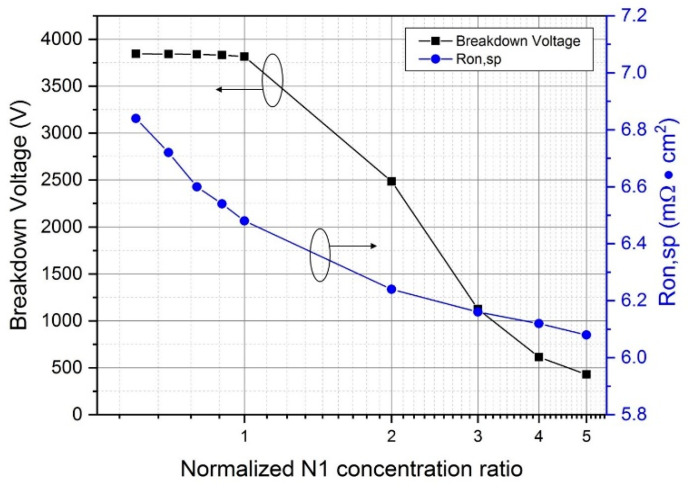
Relationship between different N1 epitaxial layer concentration ratios and the breakdown voltage and R_on,sp_.

**Figure 5:**
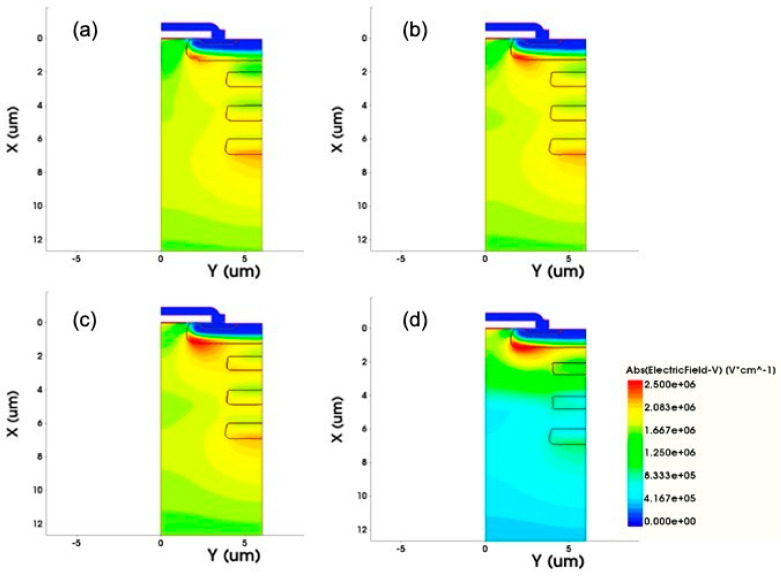
Electric field distributions of different N1 epitaxial layer concentrations: normalized N1 concentration ratio of (**a**) 0.6, (**b**) 0.8, (**c**) 1, (**d**) 3.

The concentration and thickness of the epitaxial layer N1 are the key points in the design of DC-FSJ MOSFET because the concentration directly affects the breakdown voltage and R_on,sp_ of the device. The normalized N1 value of 1.0 means that the origin N1 concentration reaches the highest reverse breakdown and it is higher than the concentration of N2. Seen from Figure 4, when the N1 epitaxial layer concentration ratio exceeds 2, the R_on,sp_ significantly decreases but the breakdown voltage dramatically drops. It can be seen from Figure 5 when the N1 epitaxial layer concentration is too high and cannot balance with the floating P-type concentration, the electric field will be crowded around the P-well area, causing the premature breakdown. As the N1 concentration fades, the electric field gradually concentrates at the bottom of the floating P-type structure. When the N1 epitaxial layer concentration and the floating P-type concentrations are compensated, the electric field is distributed more uniformly in the drift region. 

### 3.3. Thickness of N1 Epitaxial Layer

**Figure 6:**
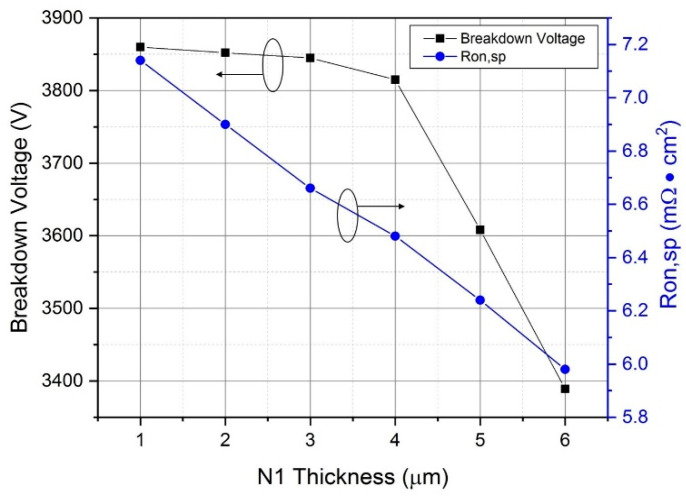
Relationship of different N1 epitaxial layer thicknesses and breakdown voltage and R_on,sp_.

**Figure 7:**
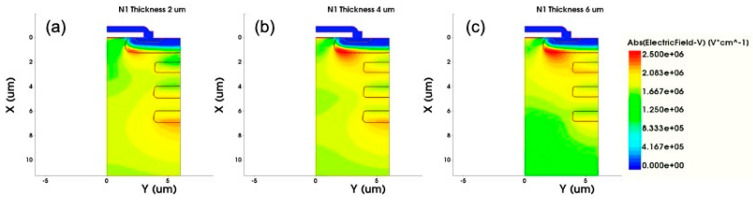
The electric field distribution diagrams of different N1 epitaxial layer thicknesses (**a**) 2 μm, (**b**) 4 μm, (**c**) 6 μm.

The thickness of the N1 epitaxial layer determines the relative position of the epitaxial layer boundary with respect to the floating P-type structure. From Figure 6, when the N1 epitaxial layer is thicker, the R_on,sp_ decreases linearly. In addition, when the thickness is less than 4 μm, the bottom of the N1 epitaxial layer is slightly above the bottom of the second floating P-type structure, which will not greatly affect the breakdown voltage of the MOSFET. Instead, when the thickness is greater than 4 μm, the breakdown voltage will drop significantly. The main reason is when the bottom of the N1 epitaxial layer exceeds the bottom of the second floating P-type structure, the electric field between the first and second floating P-structures becomes discontinuous. Furthermore, the N2 epitaxial layer mainly relies on the first floating P-type structure to extend the electric field, which mainly determines the breakdown voltage of the device as shown in Figure 7. Therefore, the thicker N1 epitaxial layer and the first floating P-type structure cannot evenly spread the electric field towards to the N2 epitaxial layer, leading to the lower breakdown voltage. Because of the thicker N1 epitaxial layer, the N-type concentration is greater than that of the P-type region, which means the electric field is easy to gather at the bottom of the P-well region. Therefore, a worse charge balance is achieved, and the breakdown voltage of the device decreases. Thus, when designing a DC-FSJ MOSFET, the thickness of the N1 epitaxial layer and the charge balance theory need to be considered together. 

### 3.4. Floating P-Type Structures in the Different Epitaxial Layers in the Drift Region

**Figure 8:**
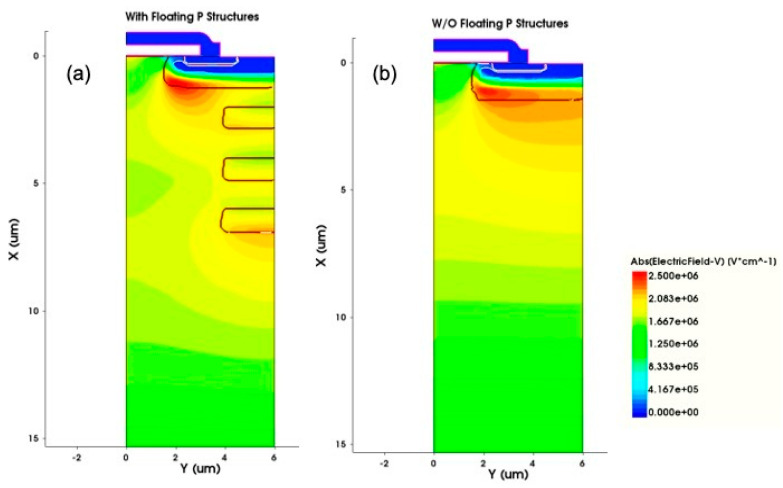
Comparison of the electric field distribution with and without the floating P-type structures (**a**) with floating P structures, (**b**) without floating P structures.

A traditional power MOSFET that uses different concentrations of the epitaxial layers will cause the dramatic drop of the breakdown voltage, because the junction of the P-well structure and the high-concentration N1 epitaxial layer is prone to inducing the high electric field and then collapsing prematurely. The floating P-type structure compensates the N1 epitaxial layer concentration and spreads out the electric field evenly so that the device can reach a higher breakdown voltage, as shown in Figure 8.

## 4. Comparison of Electrical Properties

**Figure 9:**
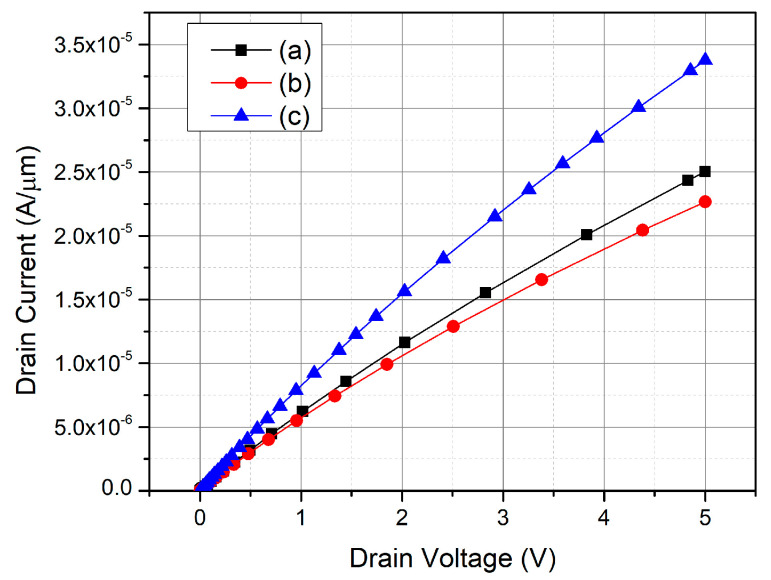
Forward I_d_-V_d_ diagram of the three structures (**a**) traditional vertical MOSFET, (**b**) FSJ MOSFET, (**c**) DC-FSJ MOSFET.

**Figure 10:**
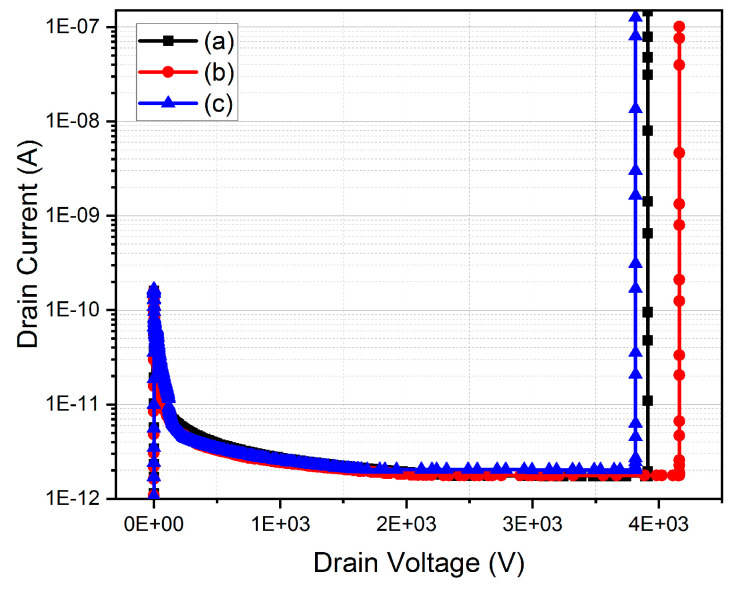
Reverse I_d_-V_d_ diagram of the three structures (**a**) traditional vertical MOSFET, (**b**) FSJ MOSFET, (**c**) DC-FSJ MOSFET.

According to the simulation results in Table 1, Figure 9 and Figure 10, the reverse breakdown voltage of the traditional vertical MOSFET is 3912 V, and R_on,sp_ is 8.66 mΩ·cm^2^; the MOSFET with floating P-type structures can extend the electric field from the P-well region to the deeper area of the drift region and then increase the breakdown voltage. In this work, the breakdown voltage can be increased up to 4162 V. However, when the FSJ-MOSFET is in the forward conduction, due to the floating structures, the area that the current flows through is narrow and the R_on,sp_ becomes the highest among these three structures (about 9.12 mΩ·cm^2^). Therefore, the N1 epitaxial layer with the higher concentration is added to form DC-FSJ MOSFET. When reaching the charge balance, the DC-FSJ MOSFET can maintain a breakdown voltage of 3800 V and reduce the R_on,sp_ to 6.5 mΩ·cm^2^. The BFOM of DC-FSJ MOSFET increases by 27% compared with the traditional vertical MOSFET; and increases by 18% compared with the BFOM of the FSJ MOSFET. The N1 epitaxial layer of DC-FSJ MOSFET can reduce the area of depletion generated by the floating P-type structures in the forward bias and then reduce the current crowding in the JFET region. The uncertainty error on the floating P-type structures should be the precision of the depth and the concentration of the floating P-type structures. If the desired depth and concentration are not achieved, the breakdown voltage will be significantly reduced and the specific on-resistance will be increased.

## 5. Conclusions

The proposed 3.3 kV 4H-SiC DC-FSJ MOSFET structure is formed by using the multiple epitaxial growth, floating P-type structures and the epitaxial layers with different concentrations. Not only can it reach the desired breakdown voltage but it also greatly reduces the R_on,sp_. Under the same conditions, the BFOM of DC-FSJ MOSFET increases by 27% and 18% compared with the traditional vertical MOSFET and the FSJ MOSFET, respectively. The R_on,sp_ also reduces by about 25% compared with the traditional vertical MOSFET.

## Figures and Tables

**Table 1:** Comparison of the characteristics of the three MOSFET structures.

Parameter	Traditional	FSJ	DC-FSJ
Thickness (μm)	30	30	26
R_on,sp_(mΩ·cm^2^)	8.66	9.12	6.5
BV (V)	3912	4162	3815
V_th_ (V)	3.13	3.35	3.29
BFOM (MW/cm^2^)	1767	1899	2239
